# Effects of Smoking, Drinking, and Urban Environment on Obesity in Seoul, Korea

**Published:** 2020-02

**Authors:** Haejung CHUN

**Affiliations:** 1 Graduate School of Business Administration, Sangmyung University, 20 Hongjimun 2-gil, Jongno-gu, Seoul, Korea

**Keywords:** Urban environment, Obesity, Spatiotemporal autoregressive model, Smoking, Housing price

## Abstract

**Background:**

The level of obesity is related to spatial characteristics around the individual. The objective of this study was to empirically analyze the effect of smoking, drinking, and urban environment on obesity in community residents.

**Methods:**

This study was conducted for empirical analysis using Ordinary Least Squares (OLS) model not considering time or space-effects, Temporal Autoregressive (TAR) model considering time-effect only, Spatial Autoregressive (SAR) model considering space-model only, and STAR model considering both time and space effects. This study covered 25 autonomous districts in Seoul City, South Korea in terms of space and from 2009 to 2014 in terms of time.

**Results:**

The STAR model yielded an adjusted R square higher than that from OLS, TAR, or SAR model. Empirical results from the STAR model showed significantly positive (+) effects of the ratio of dependent elders, ratio of smokers, ratio of drinkers, and areas of retail floor space on obesity. In contrast, effects of length of bicycle road and the amount of collected local tax on obesity were negative (−) with statistical significance.

**Conclusion:**

Smoking and drinking rate and the length of bicycle road can contribute to personal obesity.

## Introduction

The risk of obesity can reduce human lifespan by approximately 2∼5 years within the next 50 years (1). Recently, along with studies reporting the effect of urban environments on various kinds of diseases such as obesity, depression, heart diseases, diabetes mellitus, and cancers, academic interest in health improvement through urban planning and developing related policies has been growing (2). The sprawling increase of vehicle oriented traffic patterns, environments for pedestrians and bicycles, and reduction in green area have been identified as important factors that can worsen the physical health of individuals (3–13).

To examine the relationship between urban environment and obesity or health, various theories such as ‘Social Ecology Theory’, ‘Behavioral Model of Environment’, and ‘Social Determinants of Health Model’ have been employed. The ‘social ecology theory’ is based on a medical perspective. Rather than determining the health of individuals according to the presence of diseases, it takes biological, behavioral, and environmental characteristics of individuals into account to identify comprehensive factors underlying diseases in individuals (14, 15). The ‘behavioral model of environment’ takes environmental factors affecting individual exercise behaviors such as walking and bicycling into account to identify exercising behaviors and movement paths using starting point, destination, or attractiveness of neighborhood area (16, 17). The ‘social determinants of health model’ considers the effect of physical and social environment in cities on individuals’ health beyond the effect of medical factors (18, 19). In this study, these theories were taken into account to determine variables.

The level of obesity and the health of individuals are related to spatial characteristics around individuals. Through spatial interaction or spatial dependence, the level of obesity or health in certain area may rise or fall. If a model is unable to reflect spatial characteristics, then the explanatory power for results of analyzing determinants of the level of health in certain spaces would be degraded. This may necessitate a model of spatial econometrics that takes spatial correlation into account. Therefore, this study was conducted by applying a Spatiotemporal Autoregressive (STAR) model capable of taking both spatial and temporal effects into account for analyses.

The objective of this study was to quantitatively analyze the effect of drinking, smoking, and urban environment on obesity in community residents by using the spatial econometric model. Results of this study can be used as basic data to suggest divergent politic alternatives to reduce obesity rate for healthy city in terms of future urban planning.

## Methods

The STAR model was employed in this study for empirical analyses of the effect of urban environments on obesity. As a dependent variable, body mass index (BMI) reflecting the degree of obesity in individuals was taken. As independent variables, ratio of dependent elders, ratio of smokers, and ratio of drinkers were taken as those representing individuals’ characteristics. Housing price index, ratio of area of urbanization, length of all roads, length of all bicycle roads, area of parks, and area of retail floor space were used as physical environmental variables. In addition to these independent variables, the amount of local taxes collected and the number of recipients of national basic living allowance from the government were selected as proxy variables of regional economy.

### Model

The following spatiotemporal autoregressive process was assumed (20).

[1] (I−W)Y=Xβ+∈

where Y is a vector of N ×1 observations of dependent variables, X represents the matrix of N×*k* observations of independent variables, *β* is a vector of *k*×1 parameters, and *ε* denotes a vector of N×1 error terms, of which mean is 0 and variance is *σ*^2^.

Orthogonal elements of the spatiotemporal weight matrix *W* are all 0. Additionally, nonnegative elements are assumed wherein linear filters (of which a value of 1 for the sum of each column in *W*) are assumed. With the assumption of temporal observations, the structure of the matrix is that of a strictly lower triangular matrix. The matrix *W* can be decomposed into matrixes *S* and *T* to define the spatial and temporal relationship among previous observations as expressed in the following equation [2].

[2] $$W{=}_{\rho S}S{+}_{\rho T}T{+}_{\rho ST}ST{+}_{\rho TS}TS$$

Here, *ϱ****_S_*** and *ϱ* ***_T_*** are autoregressive parameters of space and time for which absolute values are assumed to be less than or equal to 1. That is, no interaction between spatial and temporal effects is assumed.

In general, *ST* and *TS* are not equal because results obtained from spatial filtering followed by temporal filtering might be different from those obtained from temporal filtering followed by spatial filtering.

Prior to model estimation, all parameters representing the spatiotemporal effect of entire samples should be constructed. In the actual process of model estimation, some initial observations should be excluded from the value of such parameters because estimation employing initially selected values may generate different results.

Application of the OLS method to spatiotemporal autoregressive models can generates bias. Therefore, instrumental variables estimation (IVE), maximum likelihood estimation (MLE), and two-stage least square estimation (2SLS) were employed to correct the bias. In this study, the MLE method was used for model estimation. By assuming that error terms are normally distributed, the log likelihood function can be obtained as expressed in the following equation [3].

[3] $$\text{ln}L=-\frac{N}{2}\text{ln}(2\pi )-\frac{N}{2}\text{ln}\sigma^{2}+\text{ln}|I-W|-\frac{1}{2}\frac{e\prime e}{\sigma^{2}}$$

From equation [3], the equation *e* = (*I* – *W*)Y – X*β* can be obtained, where |*I* – *W*| denotes the Jacobian that enables the value of integrated probability density function to be 1 in a process that converts unobserved *ε* into observable Y. In this paper, owing to characteristics of *S* and *T*, the lower triangular matrix has orthogonal element value of 0. Matrixes *ST* and *TS* are lower triangular matrixes wherein orthogonal elements are all 0. Thus, if we assume *I* – *W* = (*I*–*_ρS_S*)(*I*–*_ρT_T*), then the following equation [4] can be obtained.

[4] $$\text{ln}L=-\frac{N}{2}\text{ln}(2\pi )-\frac{N}{2}\text{ln}\sigma^{2}+\text{ln}|I{-}_{\rho S}S|-\frac{1}{2}\frac{e\prime e}{\sigma^{2}}$$

For model estimation of parameters using the MLE method, the determinant of (I–*_ρS_S*) needs to be calculated. However, with vast amounts of spatiotemporal data, the calculation process would be troublesome. Therefore, in this paper, equation $\left|I{-}_{\rho S}S|=\sum \text{ln}(1_{\delta S}\lambda_{i}\right)$ was used (21).

## Results

The effects of urban environment on obesity in regional residents were empirically analyzed by employing OLS, SAR, TAR, and STAR models.

As a dependent variable in Table 1, the ratio of people with BMI equal to or over 25 was employed to distinguish the ratio of obese people. Collected amount of local taxes and the number of recipients of national basic living allowance from the government were also taken as independent variables, serving as proxy variables to represent the regional economy. The spatial scope had 25 districts in Seoul while the temporal scope was from 2009 to 2014. Due to differences in units of collected data, some of these raw data collected were changed into log values for convenience of analyses and variance stabilization.

**Table 1:** Descriptions of variables used for the prediction of obesity

***Types of Variables***		***Variables***	***Descriptions***	***Units***	***Sources***
Dependent	Variable	Ratio of obese people	Ratio of people of respective BMI ≥ 25	%	The Seoul Statistics
Independent Variables	Individual Characteristics	Ratio of dependent elders	Ratio of dependent elders	%	
		Ratio of smokers	Current Ratio of Smokers	%	
		Ratio of drinkers	Ratio of people drunken more than once in a month during past 1 year	%	
	Physical Urban Environment	Housing Price	Actual Apartment Transaction Price Index	Jan. 2006 = 100	
		Ratio of area of urbanization	Ratio of the Area of Urbanization	%	
		Length of Roads	Length of All Roads (log)	km	
		Length of Bicycle Roads	Length of All Bicycle Roads (log)	km	
		Area of Parks	Per Capita Area of Parks (log)	m^2^	
		Area of Retail Floor Space	Total Areas of Retail Floor Space (log)	m^2^	
	Social Urban Environment	Amount of Local Taxes Collected	Amount of Local Taxes Collected (log)	Million KRW	
		Number of Recipients of National Basic Living owance	Number of Recipients of National Basic Living Allowance (log)	Number of people	

**Fig. 1:**
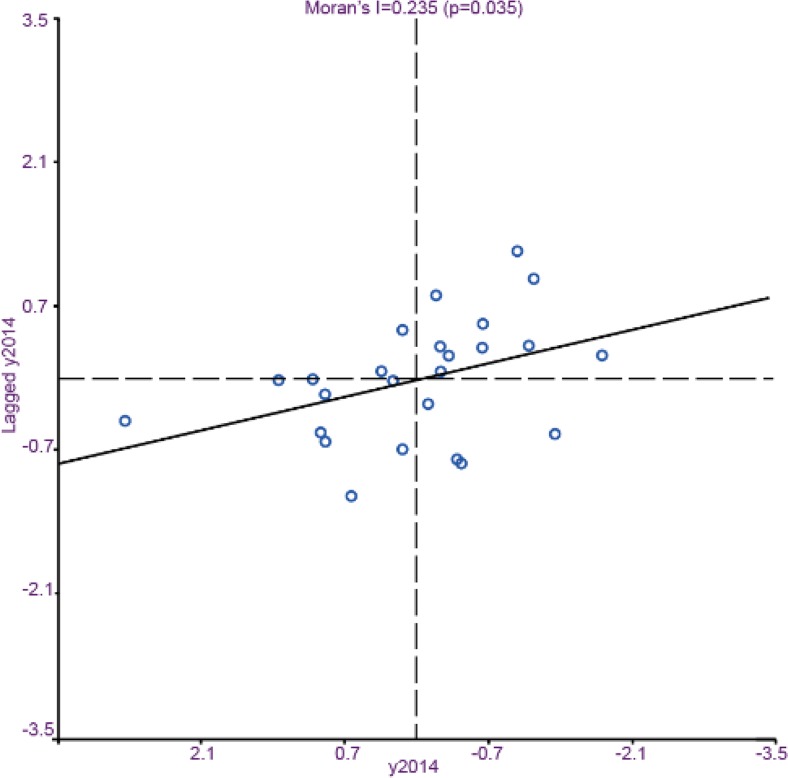
Moran's I Test of BMI, 2014

Descriptive statistics for each variable is summarized in Table 2. Spatial correlations in Seoul were tested with values of Moran’s I. The ratio of obese people was found to have a positive (+) spatial correlation as shown in Fig. 1.

**Table 2:** Basic Statistics of Variables used for the Prediction of Obesity

***Types of Variables***		***Variables***	***Mean***	***Standard Deviation***	***Minimum Value***	***Maximum Value***
Dependent	Variable	Ratio of obese people	22.86	2.48	16.10	28.40
Independent Variables	Individual Characteristics	Ratio of dependent elders	14.03	2.53	9.10	20.30
		Ratio of smokers	22.63	2.35	14.70	27.70
		Ratio of drinkers	59.74	3.29	43.60	64.80
	Physical Urban Environment	Housing Price Index	136.03	15.35	97.90	182.30
		Ratio of area of urbanization	64.95	18.21	37.33	100.10
		Length of Roads	326.48	96.13	114.00	627.00
		Length of Bicycle Roads	20.14	19.65	0.50	109.00
		Area of Parks	10.78	9.47	1.64	39.76
		Area of Retail Floor Space	182952.90	150287.20	16299.00	679936.00
	Social Urban Environment	Amount of Local Taxes Collection	479840.80	409179.10	158718.00	2212952.00
		Number of Recipients of National Basic Living Allowance	8268.33	4306.73	2819.00	22848.00

**Fig. 2:**
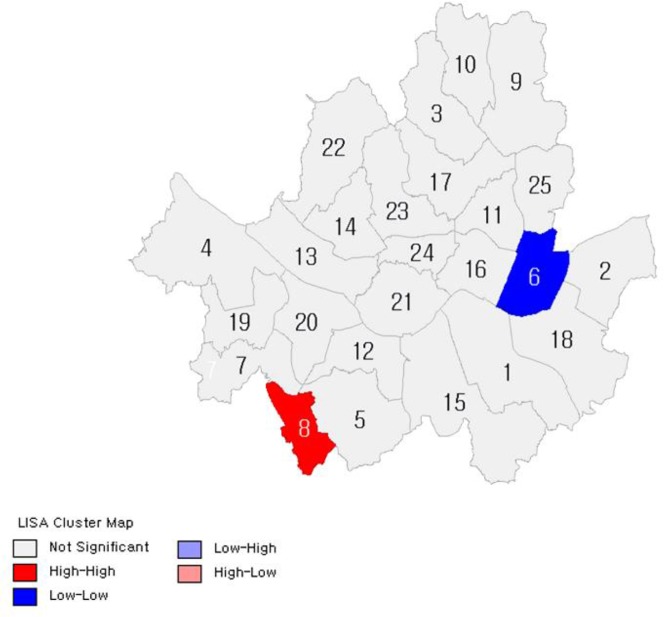
Results of Hot Spot Analyses on the Ratio of Obese People(t=2009) Note: 1 indicates Gangnam-gu; 2, Gangdong-gu; 3, Gangbuk-gu; 4, Gangseo-gu; 5, Gwanak-gu; 6, Gwangjin-gu; 7, Guro-gu; 8, Geumcheon-gu; 9, Nowon-gu; 10, Dobong-gu; 11, Dongdaemun-gu; 12, Dongjak-gu; 13, Mapo-gu; 14, Seodaemun-gu; 15, Seocho-gu; 16, Seongdong-gu; 17, Seongbuk-gu; 18, Songpa-gu; 19, Yangcheon-gu; 20, Yeongdeungpo-gu; 21, Yongsan-gu; 22, Eunpyeong-gu; 23, Jongno-gu; 24, Jung-gu; 25, Jungnang-gu

**Fig. 3:**
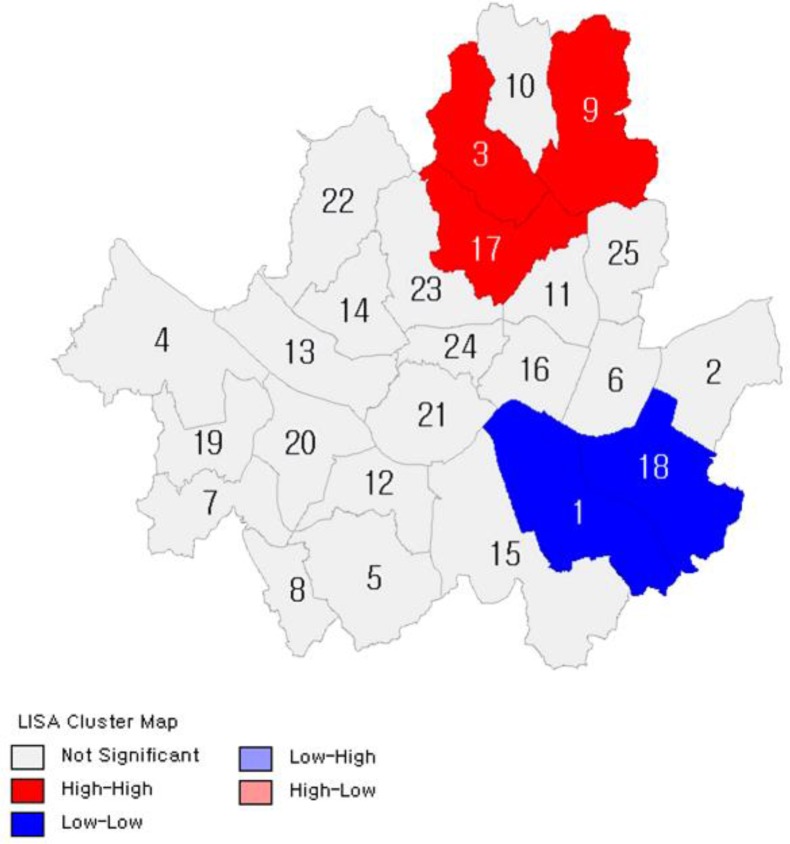
Results of Hot Spot Analyses on the Ratio of Obese People(t=2010) Note: 1 indicates Gangnam-gu; 2, Gangdong-gu; 3, Gangbuk-gu; 4, Gangseo-gu; 5, Gwanak-gu; 6, Gwangjin-gu; 7, Guro-gu; 8, Geumcheon-gu; 9, Nowon-gu; 10, Dobong-gu; 11, Dongdaemun-gu; 12, Dongjak-gu; 13, Mapo-gu; 14, Seodaemun-gu; 15, Seocho-gu; 16, Seongdong-gu; 17, Seongbuk-gu; 18, Songpa-gu; 19, Yangcheon-gu; 20, Yeongdeungpo-gu; 21, Yongsan-gu; 22, Eunpyeong-gu; 23, Jongno-gu; 24, Jung-gu; 25, Jungnang-gu

**Fig. 4:**
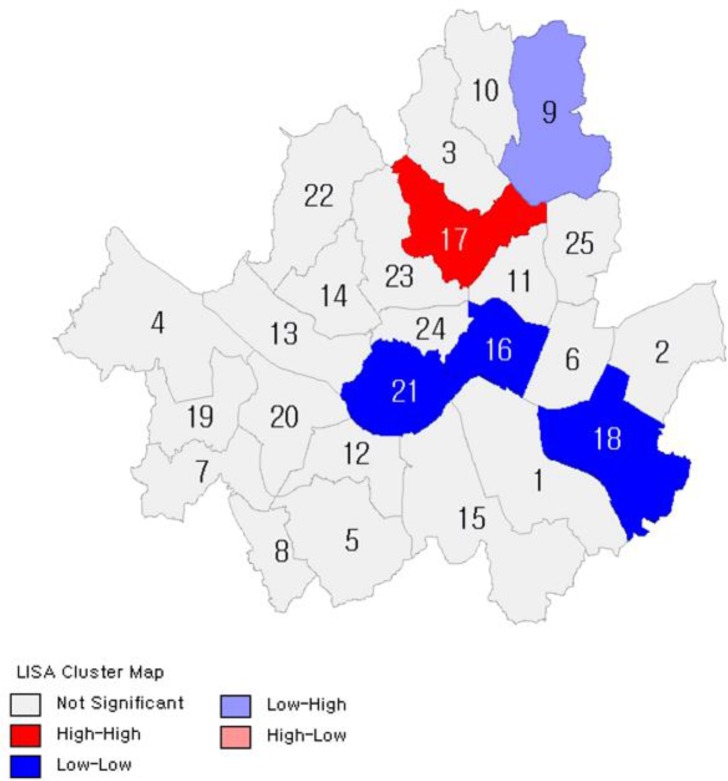
Results of Hot Spot Analyses on the Ratio of Obese People(t=2011) Note: 1 indicates Gangnam-gu; 2, Gangdong-gu; 3, Gangbuk-gu; 4, Gangseo-gu; 5, Gwanak-gu; 6, Gwangjin-gu; 7, Guro-gu; 8, Geumcheon-gu; 9, Nowon-gu; 10, Dobong-gu; 11, Dongdaemun-gu; 12, Dongjak-gu; 13, Mapo-gu; 14, Seodaemun-gu; 15, Seocho-gu; 16, Seongdong-gu; 17, Seongbuk-gu; 18, Songpa-gu; 19, Yangcheon-gu; 20, Yeongdeungpo-gu; 21, Yongsan-gu; 22, Eunpyeong-gu; 23, Jongno-gu; 24, Jung-gu; 25, Jungnang-gu

**Fig. 5:**
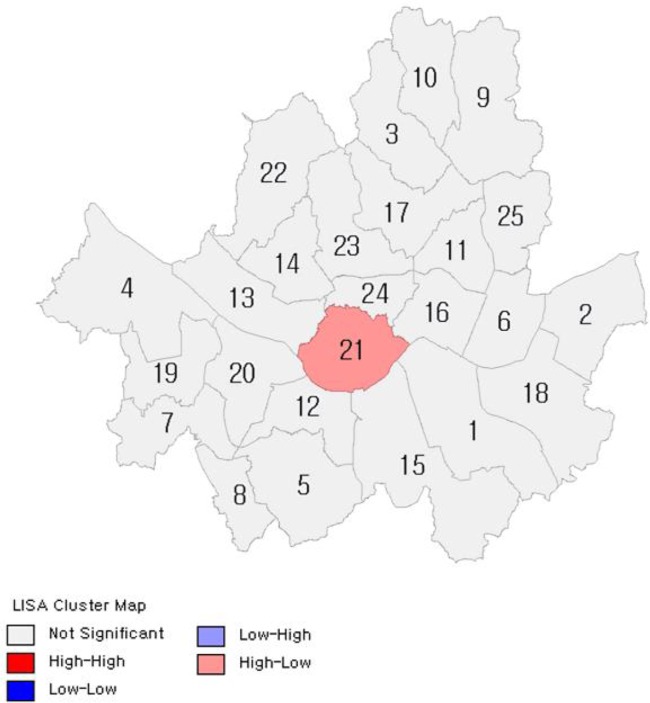
Results of Hot Spot Analyses on the Ratio of Obese People(t=2012) Note: 1 indicates Gangnam-gu; 2, Gangdong-gu; 3, Gangbuk-gu; 4, Gangseo-gu; 5, Gwanak-gu; 6, Gwangjin-gu; 7, Guro-gu; 8, Geumcheon-gu; 9, Nowon-gu; 10, Dobong-gu; 11, Dongdaemun-gu; 12, Dongjak-gu; 13, Mapo-gu; 14, Seodaemun-gu; 15, Seocho-gu; 16, Seongdong-gu; 17, Seongbuk-gu; 18, Songpa-gu; 19, Yangcheon-gu; 20, Yeongdeungpo-gu; 21, Yongsan-gu; 22, Eunpyeong-gu; 23, Jongno-gu; 24, Jung-gu; 25, Jungnang-gu

**Fig. 6:**
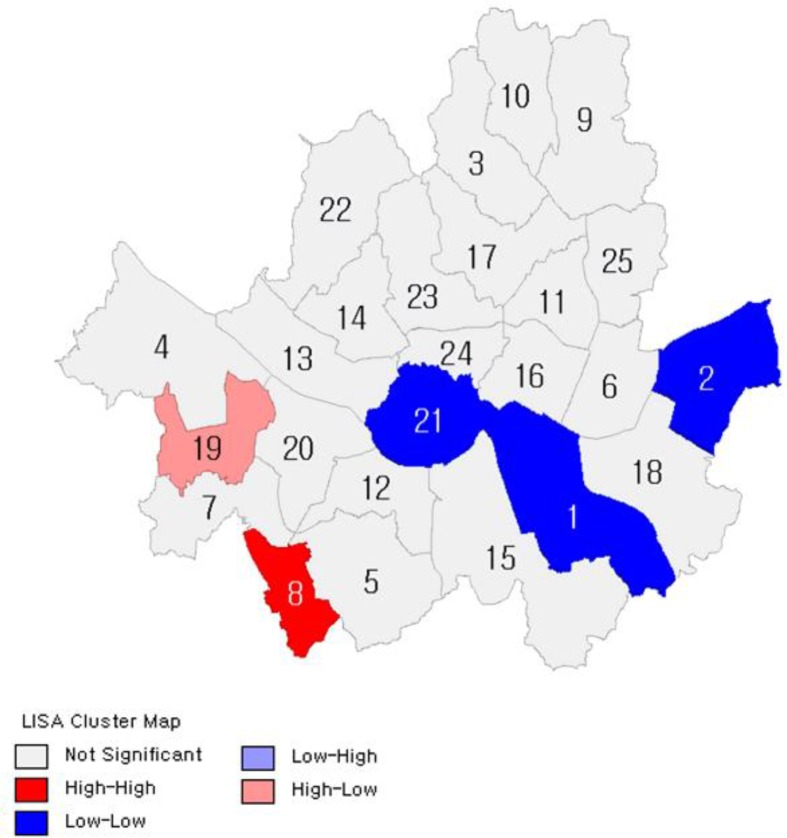
Results of Hot Spot Analyses on the Ratio of Obese People (t=2013) Note: 1 indicates Gangnam-gu; 2, Gangdong-gu; 3, Gangbuk-gu; 4, Gangseo-gu; 5, Gwanak-gu; 6, Gwangjin-gu; 7, Guro-gu; 8, Geumcheon-gu; 9, Nowon-gu; 10, Dobong-gu; 11, Dongdaemun-gu; 12, Dongjak-gu; 13, Mapo-gu; 14, Seodaemun-gu; 15, Seocho-gu; 16, Seongdong-gu; 17, Seongbuk-gu; 18, Songpa-gu; 19, Yangcheon-gu; 20, Yeongdeungpo-gu; 21, Yongsan-gu; 22, Eunpyeong-gu; 23, Jongno-gu; 24, Jung-gu; 25, Jungnang-gu

**Fig. 7:**
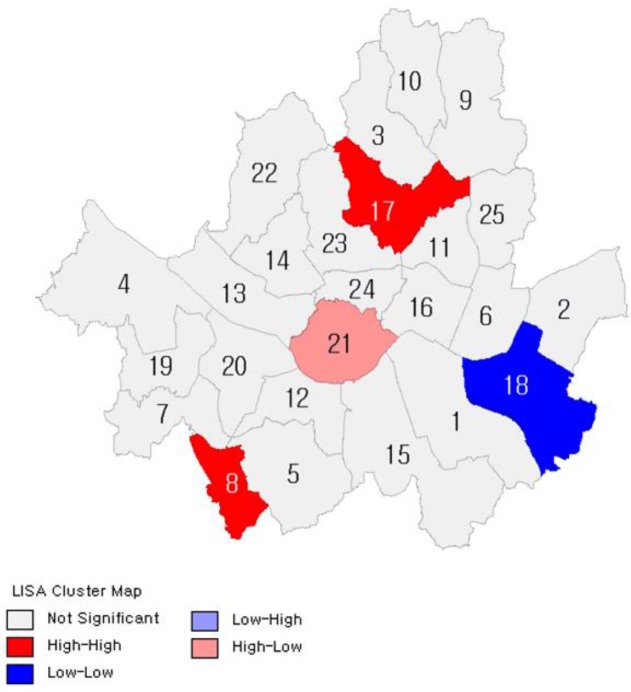
Results of Hot Spot Analyses on the Ratio of Obese People(t=2014) Note: 1 indicates Gangnam-gu; 2, Gangdong-gu; 3, Gangbuk-gu; 4, Gangseo-gu; 5, Gwanak-gu; 6, Gwangjin-gu; 7, Guro-gu; 8, Geumcheongu; 9, Nowon-gu; 10, Dobong-gu; 11, Dongdaemun-gu; 12, Dongjak-gu; 13, Mapo-gu; 14, Seodaemun-gu; 15, Seocho-gu; 16, Seongdong-gu; 17, Seongbuk-gu; 18, Songpa-gu; 19, Yangcheon-gu; 20, Yeongdeungpo-gu; 21, Yongsan-gu; 22, Eunpyeong-gu; 23, Jongno-gu; 24, Jung-gu; 25, Jungnang-gu

Figures 2–7 show the results of hot spot analyses of annual ratio of obese people in Seoul from 2009 to 2014. The statistical significance of the result of hot spot analyses was determined by 95% level of significance through z-test.

Results of empirical analyses employing OLS, TAR, SAR, and STAR models are summarized in Table 3. The estimation with the STAR model yielded adjusted R square of 0.621, which was approximately 14% higher than that with the OLS model. Its RMSE was 1.682, approximately 10% less than that with the OLS model. Since the STAR model had the highest estimation accuracy, results of the estimation from the STAR model will be described.

**Table 3:** Results of Empirical Analyses

***Variables***	***OLS***	***TAR***	***SAR***	***STAR***
Constant Terms	33.555 (9.256)^**^	32.691 (9.501)	29.492 (13.323)^**^	29.761 (9.216)^**^
Ratio of dependent elders	0.284 (0.078)^*^	0.127 (0.108)	0.067 (0.150)	0.309 (0.077)^**^
Ratio of smokers	0.199 (0.081)^**^	0.060 (0.101)	0.098 (0.119)	0.189 (0.077)^*^
Ratio of drinkers	0.175 (0.049)	0.142 (0.053)^**^	0.351 (0.090)^**^	0.184 (0.047)^**^
Housing Price Index	−0.019 (0.016)	−0.007 (0.021)	−0.029 (0.030)	−0.017 (0.015)
Ratio of area of urbanization	−0.002 (0.012)	0.000 (0.012)	−0.047 (0.016)^**^	−0.001 (0.011)
Length of Roads	0.043 (0.638)	0.133 (0.633)	0.257 (0.721)	0.033 (0.608)
Length of Bicycle Roads	−0.562 (0.291)	−0.536 (0.292)	−1.243 (0.487)^*^	−0.596 (0.278)^*^
Area of Parks	−0.089 (0.251)	−0.117 (0.246)	−0.632 (0.349)	−0.054 (0.240)
Area of Retail Floor Space	0.910 (0.270)^**^	0.779 (0.273)^**^	2.113 (0.400)^**^	1.023 (0.269)^**^
Amount of Local Taxes Collection	−2.540 (0.436)^**^	−2.343 (0.465)^**^	−3.700 (0.598)^**^	−2.402 (0.427)^**^
Number of Recipients of National Basic Living Allowance	0.553 (0.428)	0.295 (0.455)	−0.076 (0.577)	0.566 (0.408)
Rho	-	-	0.012 (0.008)	−0.009 (0.007)
The adjusted R squares	0.477	0.516	0.552	0.621
Log-L	−299.874	−298.876	−294.0081	−287.428
AIC	623.747	622.0163	3.207	1.878
DW	2.018	2.133	2.142	2.374
RMSE	1.862	1.824	1.794	1.682

Notes.

1) ^**^ and ^*^ denote significance level at 1% and 5%, respectively

2) Values in ( ) are those of the standard error

In the estimate from the STAR model, the ratio of dependent elders, ratio of smokers, ratio of drinkers, and total areas of retail floor space appeared to have significantly positive (+) effects on BMI. However, the length of all bicycle roads (0.278) and the amount of local taxes collected (0.427) showed significant negative (−) effects on BMI.

Effects of various individual independent variables on the ratio of obese people were as follows. A 1% increase in the ratio of dependent elders corresponded to a 0.309% increase in the ratio of obese people. A 1% increase in the ratio of smokers corresponded to a 0.189% increase in the ratio of obese people. A 1% increase in the ratio of drinkers corresponded to a 0.184% increase in the ratio of obese people. A 1% increase in the length of bicycle roads corresponded to a 0.006% decrease in the ratio of obese people whereas a 1% increase in the total area of retail floor space corresponded to a 0.010% increase in the ratio of obese people. A 1% increase in the amount of local taxes collected corresponded to a 0.024% decrease in the ratio of obese people.

## Discussion

First, since ratio of smokers and ratio of drinkers appeared to have significant positive (+) effects on the ratio of obese people, regulation of facilities that distribute such products may be reflected in the establishment of urban policies to improve the level of public health and quality of life.

Second, the length of roads designated for bicycles appeared to have significantly negative (−) effects on the ratio of obese people whereas the length of roads designated for vehicles showed insignificant but positive (+) effects. This encourages governments to take construction or extension of roads for bicycles into account, with additional road safety installations and pedestrian lanes during urban planning. Dedicated streets for bicycles or installation of bicycle depositories in public transportation stations including subways and city bus lines are presumed to facilitate more cycling by residents.

Third, statistically significant negative (−) effects of the amount of local taxes collected and significant positive (+) effects of the number of recipients of national basic living allowance on the ratio of obese people suggest that higher and lower levels of income may respectively cause the ratio of obese people to increase and decrease, respectively. This also suggests that the government should implement various direct and indirect policies to support the low-income bracket to reduce the ratio of obese people.

In summary, to increase quality of life in community residents and reduce obesity rate, the government needs to establish institutional tool for policy-planning and policy-implementing by reflecting suggestions derived from this study and move forward to consider politic practicability by encouraging resident participation at divergent level and building a cooperative system.

## Conclusion

Independent variables such as ratio of dependent elders, ratio of smokers, ratio of drinkers, and total areas of retail floor space appeared to have significantly positive (+) effects on the ratio of obese people whereas the length of bicycle roads and amount of local taxes collected showed significantly negative (−) effects. Although the number of recipients of national basic living allowance and the length of roads exhibited positive (+) effects on the ratio of obese people whereas housing price index, ratio of area of urbanization, and per capita area of parks showed.

## Ethical considerations

Ethical issues (Including plagiarism, informed consent, misconduct, data fabrication and/or falsification, double publication and/or submission, redundancy, etc.) have been completely observed by the author.
